# A Diagnosis Not to Miss: A Case of Fitz-Hugh-Curtis Syndrome

**DOI:** 10.1155/2022/1185314

**Published:** 2022-10-10

**Authors:** Jenny Choy, Vikas Sethi, Jose Mosco-Guzman, Thomas Hoffman, Weston Connelly

**Affiliations:** HCA Healthcare, USF Morsani College of Medicine, GME, HCA Florida Largo Hospital, 201 14th St SW, Largo, FL 33770, USA

## Abstract

Fitz-Hugh-Curtis syndrome is a rare disease attributed to complications of pelvic inflammatory disease secondary to chlamydia or gonorrhea infections. Patients generally complain of vague abdominal pain that is often acute in onset, with or without genitourinary complaints. We present a case of Fitz-Hugh-Curtis syndrome with a young female who presents with a complaint of right upper quadrant abdominal pain for 2 months' duration. She initially had no genitourinary complaints. She underwent a diagnostic laparoscopy and cholecystectomy during which adhesions from the lateral liver to the abdominal wall were visualized. The cholecystectomy did not relieve her pain. She later complained of abnormal vaginal bleeding for 15 days one month prior to her admission, unbeknownst to the medical team on admission. A chlamydia DNA probe test was positive, and the diagnosis of Fitz-Hugh-Curtis syndrome was made.

## 1. Introduction

Fitz-Hugh-Curtis syndrome (FHCS), also known as perihepatitis, is a rare complication of pelvic inflammatory disease (PID) that occurs in 10% of women with PID, characterized by inflammation of the liver capsule with associated abdominal pain [1, 2]. It is generally associated with PID due to chlamydia or gonococcal infections [1, 3]. It is also characterized by “violin-string adhesions” directly visualized through laparoscopy [4]. Symptoms may include fever, pleuritic pain, right upper quadrant tenderness, and/or cervical motion tenderness [1].

FHCS is often missed as a diagnosis for patients with right upper quadrant (RUQ) pain, resulting in longer lengths of stay and higher costs of care [5], and is an important differential diagnosis to consider, especially for patients presenting with RUQ pain without gallstones [6]. Murphy's sign has a high sensitivity but low specificity for cholecystitis, and it is possible to have a false-positive Murphy's sign lead to unnecessary surgery [7].

## 2. Case Presentation

A 30-year-old Caucasian female with no past medical history presented to the emergency department in early autumn of 2021 with a complaint of worsening RUQ abdominal pain for 2 months' duration. She described the pain as dull, pleuritic, and nonradiating, progressively worsening until 1 week prior to admission, when it became an excruciating stabbing pain. She denied any nausea, vomiting, or changes in her bowel habits. She was afebrile, and her vital signs were stable.

Initial laboratory tests were unremarkable. Her white blood cell count on admission was 9.4 × 10⁹/L and remained within normal limits during her hospital stay. Liver enzymes were remarkable for an uptrend in aspartate aminotransferase (AST) from 19 IU/L to 165 IU/L (normal 15–37 IU/L) and an uptrend in alanine aminotransferase (ALT) from 38 IU/L to 328 IU/L (normal 12–78 IU/L). The patient's AST maintained an uptrend from days 1 to 3 of hospitalization, increasing from 19 IU/L to 64 IU/L. The AST increased to 165 IU/L following surgery on day 4 of hospitalization, which may be indicative of an expected postoperative reaction. The ALT trend revealed a similar pattern with an initial measurement of 38 IU/L to 101 IU/L on day 3 of hospitalization and a more drastic elevation to 276 on day 4 of hospitalization following surgery. Alkaline phosphatase levels remained within normal limits during her hospital stay, with a peak value of 87 IU/L (normal 45–117 IU/L). Total bilirubin was within normal limits on initial laboratory results at 0.6 mg/dL, with an increase to 1.5 mg/dL following surgery.

**Figure 1:**
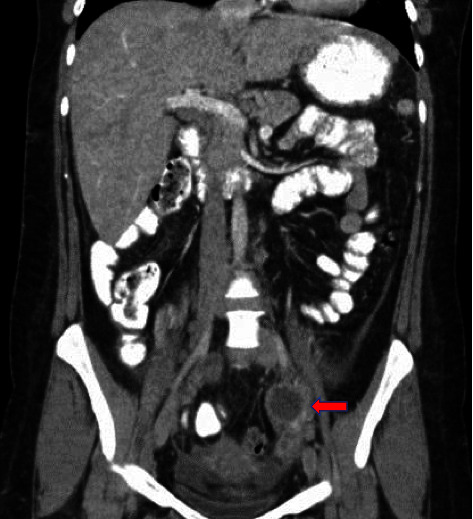
Coronal view of the lesion within the left adnexa, which contains a thin septation measuring 4.3 cm and adjacent free fluid extending to the posterior cul-de-sac and a homogeneously enlarged liver.

**Figure 2:**
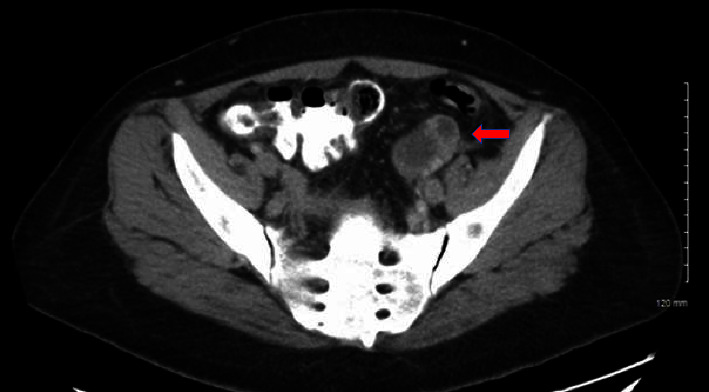
Transverse view of the lesion within the left adnexa, which contains a thin septation measuring 4.3 cm and adjacent free fluid extending to the posterior cul-de-sac.

A right upper quadrant ultrasound revealed gallbladder sludge, mild hepatomegaly, and hepatic steatosis. A hepatobiliary iminodiacetic acid (HIDA) scan showed no evidence of cystic or common bile duct obstruction. An esophagogastroduodenoscopy (EGD) was performed, which was unremarkable. A computed tomography scan of the abdomen and pelvis revealed a homogenous enlarged liver, no gallbladder pathology, a lesion within the left adnexa that contained a thin septation measuring 4.3 cm and adjacent free fluid extending to the posterior cul-de-sac, and a lobulated area adjacent to the tip of the appendix of uncertain etiology that measured 5.2 × 4.3 cm (Figures 1 and 2).

The patient elected for a diagnostic laparoscopy and cholecystectomy due to persistent pain. On direct visualization during laparoscopy, there was inflammatory fluid in the pelvis and adhesions of the right lateral segment of the liver to the abdominal wall. She initially had pain relief after the cholecystectomy; however, the pain returned the next day. Liver enzymes were gradually increasing throughout her admission. She then complained of abnormal vaginal bleeding for 15 days one month prior to her admission. This abnormal vaginal bleeding from one month prior was unbeknownst to the medical team. She was started on ceftriaxone and doxycycline for empiric coverage. An in-house chlamydia nucleic acid amplification test obtained from a cervical swab was positive, and the diagnosis of Fitz-Hugh-Curtis syndrome was made. She was sent home with instructions to consult a gynecologist.

## 3. Discussion

FHCS is a rare disease attributed to complications of PID secondary to chlamydia or gonorrhea infections, with an incidence of approximately 4% in those presenting with mild to moderate PID [3]. It is a relatively new syndrome, first mentioned by Stajano in 1920 and further described by Curtis in 1930 with direct visualization of adhesions on the liver to the abdominal wall during laparoscopy in patients presenting with atypical RUQ pain attacks [5]. Curtis discovered that many of these patients had no liver or gallbladder pathology and, instead, frequently found evidence of gonococcal infection. Fitz-Hugh Jr. described similar cases, and smears of the peritoneal fluid revealed gonococci. We now know that perihepatitis can also include *Chlamydia trachomatis*, which has become the most common cause of FHCS.

It is theorized that the microorganisms spread from the genitourinary tract via spontaneous ascending infection, through lymphatic channels, and potentially through hematogenous spread. Patients with FHCS may present with fever, nausea, vomiting, RUQ abdominal pain, pelvic pain, vaginal discharge, abnormal vaginal bleeding, etc. [1]. FHCS is an important diagnosis to consider in young females presenting with RUQ pain that does not fit a cholestatic picture, and although it is even rarer in men, cases of FHCS have also been reported in men.

Evaluation of the patient should include a pregnancy test to rule out an ectopic pregnancy and to assist with antibiotic management (Table 1). Other tests to consider include a complete blood count to assess for leukocytosis, a comprehensive metabolic panel to assess hepatic function, along with chlamydia and gonorrhea probes. Liver enzymes (AST/ALT) may be normal or slightly elevated in FHCS. For this specific case, the laboratory findings of this patient revealed a slow uptrend of both AST and ALT from admission to day 3 of her hospitalization, with a more pronounced elevation after surgery on day 4 of her hospitalization. The initial elevation of the liver enzymes, in this case, may have been secondary to the perihepatitis or due to the stress of hospitalization. The more pronounced elevation of the liver enzymes following surgical intervention may have been secondary to an expected postoperative reaction.

Imaging may include computerized tomography of the abdomen and pelvis, which may reveal perihepatic enhancement, tubo-ovarian abscesses, or fluid collections. Surgical intervention such as laparoscopy may reveal the adhesions between the liver and the diaphragm and anterior abdominal wall, such as was visualized during laparoscopy in this patient with adhesions of the right lateral segment of the liver to the abdominal wall [1].

Treatment includes symptom management and antibiotics geared towards *Chlamydia trachomatis* and *Neisseria gonorrhoeae*, which may include ceftriaxone with azithromycin, or in more complicated cases, ceftriaxone, doxycycline, and metronidazole. The prognosis for FHCS is good with antibiotic treatment. Complications may include infertility, ectopic pregnancy, and rarely bowel obstruction due to the adhesions. FHCS can be a challenging diagnosis to make and should be a diagnosis to consider for patients, both men and women, with RUQ pain and a risk for chlamydia or gonorrhea.

## Figures and Tables

**Table 1:** Common features of Fitz-Hugh-Curtis syndrome, including symptoms, laboratory values, and radiographic findings [1].

Features of Fitz-Hugh-Curtis syndrome	
Common presenting symptoms	Constitutional symptoms: Fevers, chills
	Gastroenterological symptoms: Right upper quadrant abdominal pain, nausea, vomiting
	Genitourinary symptoms: Vaginal or penile discharge, lower abdominal pain

Laboratory values	Leukocytosis, however, only to a clinically significant value in 50% of patients
	Normal to slightly elevated liver enzyme values
	Positive chlamydia or gonorrhea test

Radiographic findings	Computerized tomography with increased perihepatic enhancement, pelvic fat infiltration, tubo-ovarian abscess, and fluid collection in the pelvic cavity
	Transvaginal ultrasound suggestive of pelvic inflammatory disease hydrosalpinx, pyosalpinx endometritis, tubo-ovarian abscess, oophoritis, and ectopic pregnancy

## Data Availability

Access to the data supporting the results of this case report is restricted due to patient privacy laws and the policies implemented by the institution from which this case originated.
